# Investigation of cardiorenal outcomes and incidence of genitourinary tract infection after combined SGLT2 inhibitor and ACEI/ARB use in patients with chronic kidney disease stages 3-5: A real-world retrospective cohort study in Taiwan

**DOI:** 10.7150/ijms.96969

**Published:** 2024-08-12

**Authors:** Yu-Hsuan Joni Shao, Wan-Ting Chen, Samuel Mon-Wei Yu, Liam Li-An Tsou, Yung-Ho Hsu, Mai-Szu Wu, Yung-Hsi Kao, Chu-Lin Chou, Po-Jen Hsiao

**Affiliations:** 1 Graduate Institute of Biomedical Informatics, College of Medical Science and Technology, Taipei Medical University, Taipei, Taiwan.; 2 Clinical Big Data Research Center, Taipei Medical University Hospital, Taipei, Taiwan.; 3 Health and Clinical Research Data Center, Office of Data Science, Taipei Medical University, Taipei, Taiwan.; 4 Division of Nephrology, Department of Medicine, Mount Sinai School of Medicine, New York, New York, USA.; 5 Biochemistry, Department of Chemistry, Hofstra University, Hempstead, New York, USA.; 6 Taipei Medical University-Research Center of Urology and Kidney, Taipei Medical University, Taipei, Taiwan.; 7 Division of Nephrology, Department of Internal Medicine, School of Medicine, College of Medicine, Taipei Medical University, Taipei, Taiwan.; 8 Division of Nephrology, Department of Internal Medicine, Shuang Ho Hospital, Taipei Medical University, New Taipei City, Taiwan.; 9 Division of Nephrology, Department of Internal Medicine, Hsin Kuo Min Hospital, Taipei Medical University, Taoyuan City, Taiwan.; 10 Department of Life Sciences, National Central University, Taoyuan, Taiwan.; 11 Division of Nephrology, Department of Internal Medicine, Taoyuan Armed Forces General Hospital, Taoyuan, Taiwan.; 12 Division of Nephrology, Department of Internal Medicine, Tri-Service General Hospital, National Defense Medical Center, Taipei, Taiwan.

**Keywords:** sodium‒glucose cotransporter-2 (SGLT2) inhibitor, angiotensin-converting enzyme inhibitor (ACEI)/angiotensin receptor blocker (ARB), chronic kidney disease, acute kidney injury, acute kidney disease, end-stage renal disease, cardiorenal outcome, genitourinary tract infection

## Abstract

**Background:** Sodium‒glucose cotransporter-2 (SGLT2) inhibitors offer glycaemic and cardiorenal benefits in the early stage of chronic kidney disease (CKD). However, the use of SGLT2 inhibitors may increase the risk of genitourinary tract infection (GUTI). Angiotensin-converting enzyme inhibitors (ACEIs) and angiotensin receptor blockers (ARBs) may also cause deterioration of kidney function. The long-term follow-up of cardiorenal outcomes and GUTI incidence in patients with advanced CKD receiving SGLT2 inhibitors combined with ACEIs/ARBs should be further investigated.

**Methods:** We analysed data from 5,503 patients in Taiwan's Taipei Medical University Research Database (2016-2020) who were part of a pre-end-stage renal disease (ESRD) program (CKD stages 3-5) and received ACEIs/ARBs. SGLT2 inhibitor users were matched 1:4 with nonusers on the basis of sex, CKD, and program entry duration.

**Results:** The final cohort included 205 SGLT2 inhibitor users and 820 nonusers. SGLT2 inhibitor users experienced a significant reduction in ESRD/dialysis risk (aHR = 0.35, 95% CI = 0.190.67), and SGLT2 inhibitor use was not significantly associated with acute kidney injury or acute kidney disease risk. Among SGLT2 inhibitor users, those with a history of cardiovascular disease (CVD) had greater CVD rates. Conversely, those without a CVD history had lower rates of congestive heart failure, arrhythmia, acute pulmonary oedema, and acute myocardial infarction, although the differences were not statistically significant. Notably, SGLT2 inhibitor usage was associated with a greater GUTI incidence (aHR = 1.78, 95% CI = 1.122.84) shortly after initiation, irrespective of prior GUTI history status.

**Conclusion:** Among patients with CKD stages 3-5, SGLT2 inhibitor use was linked to increased GUTI incidence, but it also significantly reduced the ESRD/dialysis risk without an episodic AKI or AKD risk. Clinical physicians should consider a personalized medicine approach by balancing GUTI episodes and cardiorenal outcomes for advanced CKD patients receiving SGLT2 inhibitors.

## Introduction

Managing glucose metabolism and selecting antihyperglycaemic agents in people with advanced chronic kidney disease (CKD) is challenging, requiring careful personalization of treatment to minimize risks 1. There are limited options for the use of antihyperglycaemic agents in patients with stage 3~5 CKD due to their reduced efficacy and increased adverse effects, potentially leading to end-stage renal disease (ESRD) that requires dialysis 2. Notably, sodium‒glucose cotransporter-2 (SGLT2) inhibitors have shown efficacy in slowing CKD progression and offer additional cardiovascular benefits, weight loss, and a reduction in blood pressure in individuals with type 2 diabetes mellitus (DM), leading to increased utilization of these agents for these purposes 3-5.

SGLT2 inhibitors are a new class of antihyperglycaemic drugs that are prescribed mainly for type 2 diabetes patients whose blood sugar remains uncontrolled despite treatment with metformin and sulfonylurea 6. Canagliflozin, the first SGLT2 inhibitor approved by the U.S. Food and Drug Administration (FDA) in March 2013, improved glycaemic control in individuals with type 2 diabetes over a 26-week period 7. However, the effectiveness of SGLT2 inhibitors diminishes as kidney function decreases, making them less suitable for later stages of CKD 3, 8, 9. Therefore, caution should be exercised when considering the use of SGLT2 inhibitors in advanced CKD patients, and specific recommendations for their use may need to be reassessed. In particular, the effectiveness of SGLT2 inhibitors in lowering glucose levels decreases as kidney function decreases, resulting in reduced efficacy in patients with advanced CKD (estimated glomerular filtration rate (eGFR) <30 mL/min) 9. Hence, the use of SGLT2 inhibitors is generally not recommended for advanced CKD patients because of their decreased effectiveness and potential adverse effects. Importantly, SGLT2 inhibitors should be used cautiously in individuals with type 1 DM and in elderly individuals, especially in more fragile individuals and individuals with a history of genitourinary tract infections (GUTIs) 10-13; moreover, there is a slight increase in the risk of lower-limb amputation. In rare cases, these medications may lead to diabetic ketoacidosis, a serious condition characterized by the accelerated breakdown of fat in the body 14-16.

Since 2006, Pay for Performance (P4P) programs in Taiwan have aimed to improve healthcare quality and prognoses for patients with CKD stages 3~5. These programs use value-based purchasing, incentives based on renal indicators, and financial rewards for adhering to clinical guidelines. Nephrologists, supported by multidisciplinary care teams, are responsible for providing recommended care and improving self-awareness education 17, 18. Before P4P programs, multidisciplinary care education without financial incentives was used to improve CKD care 19. This approach involves collaboration among healthcare professionals to establish a consensus on diagnosis, education, evaluation, and treatment 20-24. Similar multidisciplinary care education programs have been implemented globally for various conditions, including CKD, dialysis, chronic obstructive pulmonary disease (COPD), coronary artery disease, and DM 25-31. Angiotensin-converting enzyme inhibitors (ACEIs) and angiotensin receptor blockers (ARBs) may also increase the risk of kidney function deterioration and hyperkalaemia, especially in CKD patients. There are few real-world data regarding the effects of SGLT2 inhibitors in this specific population of patients with CKD stages 3-5 who receive ACEIs/ARBs and participate in pre-ESRD programs in Taiwan 32. When the use of ACEIs or ARBs to does not result in adequate control of CKD progression, this treatment strategy may be implemented, especially in patients with advanced-stage CKD. This raises the question of whether ACEIs/ARBs can be effectively combined with SGLT2 inhibitors in the management of CKD patients. To assess the efficacy and potential adverse effects of this combination therapy compared with ACEI/ARB monotherapy, we conducted a real-world retrospective cohort study. Additionally, the long-term follow-up of cardiorenal outcomes and GUTI incidence in patients with advanced CKD receiving SGLT2 inhibitors combined with ACEI/ARB treatment were further investigated. Therefore, we investigated the effects of SGLT2 inhibitors on various clinical outcomes, including acute kidney injury (AKI), acute kidney disease (AKD), ESRD with dialysis, congestive heart failure (CHF), acute pulmonary embolisms (APEs), cardiac arrhythmias, acute myocardial infarction (AMI), sepsis, and GUTIs, in patients with stage 3~5 disease. To accomplish this, we utilized the Taipei Medical University Research Database (TMURD) and focused on individuals receiving ACEI/ARB therapy who were enrolled in a pre-ESRD program in Taiwan.

## Methods

### Data source

We conducted a multicentre hospital-based cohort study using data from the TMURD, which contains electronic health records of more than 4 million patients from three affiliated teaching hospitals: Taipei Medical University Hospital (TMUH), Wan Fang Hospital (WFH), and Shuang Ho Hospital (SHH). Informed consent was waived because of the deidentification of personal information in the TMURD, and the informed consent waiver was approved by the Joint Institutional Review Board of Taipei Medical University (TMU-JIRB-N202207007).

### Study population

We enrolled 5503 patients at stages 3~5 who received ACEIs/ARBs and participated in a pre-ESRD program from 2016 to 2020 in the TMURD. Patients under 20 years of age or older than 90 years of age (*n* = 193) and those without available data after the cohort entry date (*n* = 103) were excluded. In Taiwan, the indication of SGLT2 inhibitors for treating DM was approved at the end of 2021. Among the eligible patients, those who used SGLT2 inhibitors included patients treated with dapagliflozin, canagliflozin, and empagliflozin (*n* = 215; Anatomical Therapeutic Chemical Classification System [ATC] code: A10BK) and nonusers (*n* = 5002). We matched users and nonusers by sex, CKD stage, and the time since entering the pre-ESRD program at a 1:4 ratio.

### Covariates

We considered serum data, including creatinine (SCr) and the eGFR, as covariables. The SCr and eGFR measurements obtained within 1 year before the index date were considered the baseline measures for those variables. The index date was defined as the date on which patients initiated SGLT2 inhibitor use in the exposed groups and the corresponding matching date (from the time since entering the pre-ESRD program to the index date) in controls. The following variables and associated comorbidities were recorded as covariates 1 year before the index date: sex, age, CKD stage, DM status, ischaemic heart disease (IHD) status, atrial fibrillation status, hyperlipidaemia status, ischaemic stroke status, CHF status, peripheral vascular disease (PVD) status, COPD status, chronic liver disease (CLD) status, hypertension status, and dementia status. Patients who received medications, including clopidogrel, dipyridamole, warfarin, loop diuretics, beta-2 blockers, calcium channel blockers (CCBs), antiplatelet drugs, statins, nonsteroidal anti-inflammatory drugs (NSAIDs), metformin, thiazolidinediones, sulfonylureas, alpha-glucosidase inhibitors (AGIs), dipeptidyl peptidase-4 inhibitors (DPP4is) and insulin, were defined as patients who had received medications within 1 year before the index date. All disease diagnosis codes were according to the International Classification of Disease 9th Revision, Clinical Modification (ICD-9-CM), ICD-10-CM, National Institutes of Health (NIH) procedure codes, and ATC classifications of medications (Supplementary Table 1).

### Study endpoint

The primary outcomes were AKI, AKD, ESRD with dialysis, CHF, APEs, cardiac arrhythmias, AMI, sepsis, and GUTIs. Furthermore, in this study, the primary endpoints, which represented AKI-AKD-ESRD in the progression of dialysis, were those defined in the ADQI 33 and KDIGO 34 workshops. AKI was defined as an abrupt decrease in kidney function that occurred within 7 days or less after the index date and was divided into stages 0, 1, 2, and 3 multiplied by the SCr level. AKD was described as acute or subacute damage and loss of kidney function for a duration of 7-90 days after exposure to an AKI episode and was divided into stages 0, 1, 2, and 3 multiplied by SCr levels. All the SCr levels included in the analysis were chosen on the basis of the respective highest values obtained within 0-7 and 7-90 days for AKI and AKD, respectively. ESRD with dialysis was defined as an order code by the National Health Insurance, as shown in Supplementary Table 1.

### Statistical analysis

Descriptive statistics were used to summarize demographic and baseline data. Continuous variables are presented herein as the mean with standard deviation (SD), whereas categorical variables are presented as the number of enrolees and percentage (%). The chi-square test and Student's *t* test were applied to assess differences between the two groups for categorical and continuous variables, respectively. A Cox proportional hazards regression model was used to evaluate the risk of outcomes of interest associated with SGLT2 inhibitors after controlling for demographic and clinical factors. Patients who died, were lost to follow-up, or were discharged at the end of the follow-up period before the event of interest occurred were censored. Cumulative incidence curves were plotted and tested via a logarithmic rank test. Subgroup analyses were used to evaluate whether the impact of SGLT2 inhibitors on GUTIs or CVD differed according to patients' preexisting conditions. In this study, a two-sided *p* value of < 0.05 was considered to indicate a significant difference. All analyses were performed via SAS/STAT 9.2 (SAS Institute, Cary, NC, USA).

## Results

### Baseline characteristics

**Figure 1:**
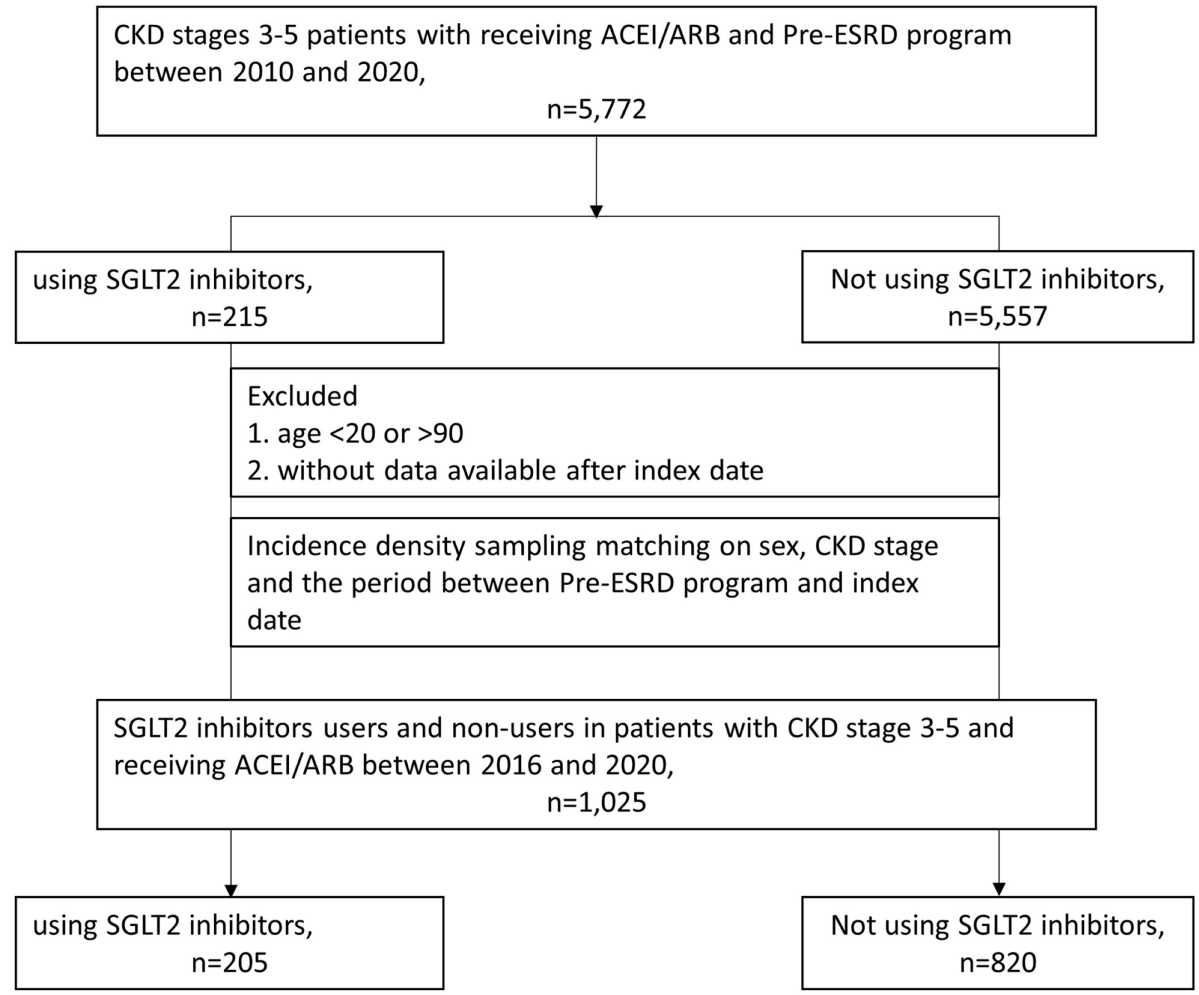
Cohort selection in patients with chronic kidney disease stages 3~5 receiving angiotensin-converting enzyme inhibitors (ACEIs)/angiotensin receptor blockers (ARBs) and participating in a pre-end-stage renal disease (ESRD) programme.

The final study included 205 patients who had received SGLT2 inhibitors and 820 patients who had not (Figure 1). During the follow-up period (median = 15.5 months), a total of 205 patients with CKD who received ACEIs/ARBs and participated in a pre-ESRD program were treated with SGLT2 inhibitors. Table 1 presents the baseline characteristics of users of SGLT2 inhibitors and their 820 sex- and stage-matched counterparts. Compared with nonusers, users of SGLT2 inhibitors were younger and had higher baseline eGFRs, ACEI/ARB ratios, Charlson comorbidity index (CCI) scores, and CHA2DS2-VASC scores. They were also more likely to have a history of heart failure, DM, IHD, and COPD than nonusers were. In addition, there were greater percentages of patients who received clopidogrel, beta-2 blockers, antiplatelet drugs, statins, and DM treatments such as metformin, thiazolidinedione, AGIs, DPP4is, and insulin among SGLT2 inhibitor users than among nonusers (Table 1).

### Risks of cardiorenal outcomes and GUTIs associated with SGLT2 inhibitor use

The incidence rates and risk estimates of outcomes associated with SGLT2 inhibitor use in patients prior to the development of ESRD are shown in Table 2. In our cohort, SGLT2 inhibitor users had a slightly lower incidence of CHF, arrhythmias, and AKD during follow-up, but the adjusted hazard ratio (aHR) did not reach statistical significance. However, SGLT2 inhibitor users had a lower incidence rate (3.78 vs. 6.59 per 100 person-years) and a significantly lower risk of ESRD/dialysis (aHR = 0.35, 95% confidence interval [CI] = 0.19~0.67) than did nonusers.

**Figure 2:**
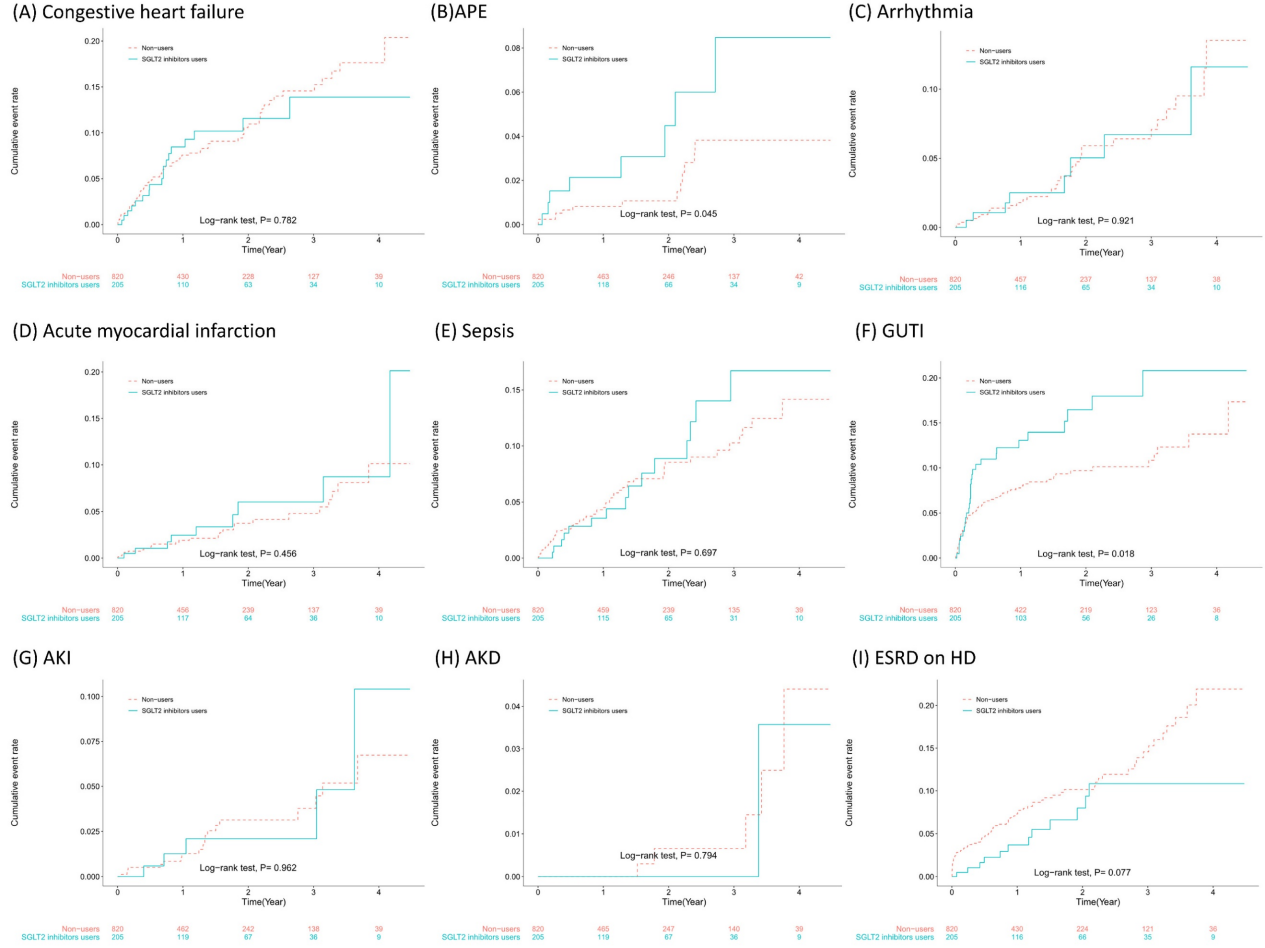
Subsequent events of sodium‒glucose cotransporter 2 (SGLT2) inhibitors in patients with chronic kidney disease stages 3~5 receiving angiotensin-converting enzyme inhibitors (ACEIs)/angiotensin receptor blockers (ARBs) and participating in a pre-end-stage renal disease (ESRD) programme.

SGLT2 inhibitor users had greater incidences of APE, AMI, sepsis, and AKI during follow-up, but the risk estimates did not reach statistical significance. SGLT2 inhibitor users had a much greater incidence (10.33 vs. 6.07 per 100 person-years) and a significantly greater risk of developing GUTIs (aHR = 1.78, 95% CI = 1.12~2.84). The cumulative incidences of the outcomes that we studied and the results of the log rank test are presented in Figure 2. As shown in Figure 2F, patients began to develop GUTIs within the first few months after initiating SGLT2 inhibitor treatment.

### Impact of preexisting conditions on the associations of SGLT2 inhibitor use with the risk of developing GUTIs and CVDs

**Figure 3:**
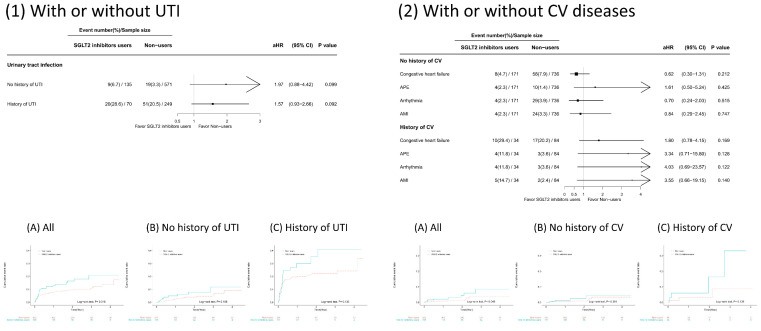
Subsequent outcomes of sodium‒glucose cotransporter 2 (SGLT2) inhibitor use in the presence or absence of a history of cardiovascular disease (CVD) and urinary tract infection (UTI) in patients with chronic kidney disease stages 3~5 receiving angiotensin-converting enzyme inhibitors (ACEIs)/angiotensin receptor blockers (ARBs) and participating in a pre-end-stage renal disease (ESRD) programme.

We conducted subgroup analyses to examine whether preexisting conditions modified the risk of developing GUTIs or CVDs associated with SGLT2 inhibitor use (Figure 3). Patients receiving SGLT2 inhibitors had a greater rate of GUTIs than nonusers did, regardless of their GUTI history. Although a greater percentage of patients developed GUTIs if they had a history of GUTIs, the risk estimate did not reach statistical significance. On the other hand, patients receiving SGLT2 inhibitors who had a history of CVD had a greater incidence of CVD than nonusers did. In contrast, patients who received SGLT2 inhibitors but did not have a CVD history had lower rates of CHF, arrhythmias, or AMI than nonusers did. However, none of these risk estimates reached statistical significance.

## Discussion

This is the first study focusing on CKD patients (stages 3~5) receiving ACEIs/ARBs in Taiwan who were enrolled in a pre-ESRD program with multidisciplinary teams to improve care and patient awareness. The major findings showed that (1) SGLT2 inhibitor use was associated with a significantly increased incidence of GUTIs (aHR = 1.78, 95% CI = 1.12~2.84), which usually occurred within months after initiation; (2) increased GUTI rates among SGLT2 inhibitor users persisted regardless of their GUTI history, with an increasing trend for patients with prior GUTIs, although not statistically significant for a prior GUTI history; (3) SGLT2 inhibitor users had a significant reduction in the risk of ESRD/dialysis (aHR = 0.35, 95% CI = 0.19~0.67), but SGLT2 inhibitor use was not associated with the risk of AKI or subsequent AKD; and (4) SGLT2 inhibitor users with a history of CVDs had higher CVD rates, whereas patients without a CVD history had lower CHF, arrhythmia, and AMI rates, although these rates did not significantly differ between patients with and without a CVD history.

Although SGLT2 inhibitors have demonstrated significant benefits for managing type 2 DM, the potential association between their use and an increased risk of developing GUTIs requires careful consideration 6. Our study suggested that the use of SGLT2 inhibitors increased the risk of developing GUTIs, especially in those with a history of GUTIs, although the trend was not statistically significant. The exact mechanism underlying this association is not yet fully understood. Hypothesized mechanisms include an association between glucose concentrations and GUTI risk, the impact of urinary pH on GUTI incidence, changes in the urinary microbiome, and immune responses in the urinary tract of SGLT2 inhibitor users 35, 36. Many studies have shown a statistically significant increase in GUTI risk among patients taking these medications 36-40, whereas other studies have not established a clear link 13, 41. It is essential to consider individual patient factors, such as age, sex, preexisting medical conditions, and overall health status, which may influence the likelihood of developing GUTIs. Physicians must strike a balance between glycaemic control and GUTI risk when prescribing SGLT2 inhibitors by considering individual patient characteristics.

However, concerns have been raised regarding the safety profile of SGLT2 inhibitors, particularly concerning their potential impacts on kidney outcomes in patients with advanced kidney disease. Several clinical trials and real-world studies have suggested potential renoprotective effects of SGLT2 inhibitors, including a reduction in albuminuria and a slower decline in the GFR 42, 43. These findings have sparked interest in their use, particularly in people with diabetes who are at risk of developing diabetic kidney disease. Moreover, FDA reports of AKI in patients using SGLT2 inhibitors have raised concerns about their safety, particularly in those with compromised renal function 44, 45. In our cohort study focused on patients with CKD stages 3~5 who received ACEIs/ARBs and participated in a pre-ESRD program, we found a trend towards a decrease in the risk of AKI and subsequent AKD progression, albeit without statistical significance. Recent meta-analyses also revealed an association between SGLT2 inhibitor use and a reduced risk of AKI 46, 47.

The long-term effects of SGLT2 inhibitors on the progression of kidney disease to ESRD are not fully understood. In this 5-year cohort study, we found a significant reduction in the risk of developing ESRD or requiring dialysis among users of SGLT2 inhibitors, which was similar to the findings of the CREDENCE trial 48. Furthermore, the use of SGLT2 inhibitors in diabetic patients without oliguria undergoing dialysis poses unique challenges. Dose adjustments and considerations for concurrent medications must be carefully managed to avoid adverse effects and optimize diabetes control. In addition to the established cardiovascular benefits of SGLT2 inhibitors, the updated data support the use of SGLT2 inhibitors to modify the risk of CKD progression and AKD, not only in patients with type 2 DM at high cardiovascular risk but also in patients with CKD or heart failure irrespective of diabetes status, primary kidney disease, or kidney function 49, 50. Ongoing research and clinical trials are continuing to explore the safety and efficacy of SGLT2 inhibitors in patients with advanced kidney disease or who are undergoing dialysis.

Notably, in our study, SGLT2 inhibitor users with a history of CVD had higher rates of CVD than those without a history of CVD did. Although the aHR did not reach statistical significance, the results suggested that such patients might have more advanced or poorly controlled disease or that SGLT2 inhibitors might not be as effective in this subgroup. Moreover, our study revealed decreased rates of AMI, CHF, and arrhythmias in SGLT2 inhibitor users without a CVD history, and while not statistically significant, these findings suggest a protective effect and cardiovascular benefits. Research on several SGLT2 inhibitors has suggested potential reductions in the atherogenic lipid profile, plaque progression, and systemic inflammation and improvements in endothelial function, which may have implications for preventing myocardial infarction. Clinical trials revealed surprising cardiovascular benefits of SGLT2 inhibitors, such as reduced cardiovascular mortality and decreased risks of major adverse cardiovascular events (MACEs) 51, 52. SGLT2 inhibitors can effectively reduce the risk of hospitalization for HF and cardiovascular death in patients with DM and CVD. The possible mechanism of action involved in promoting natriuresis and reducing cardiac workload has shown promising results in HF management 51-53. Moreover, although there is limited evidence to suggest a direct association between SGLT2 inhibitor use and arrhythmia risk, some studies have revealed a potential protective effect of these medications against atrial fibrillation and ventricular arrhythmias 53-55. As SGLT2 inhibitors are relatively new, long-term safety data are still evolving. Continued larger cohort surveillance and real-world evidence are essential to further understand the cardiovascular effects of these treatments over extended treatment periods 56-58.

This study demonstrated the effect of SGLT2 inhibitors in a real-world population with a relatively long follow-up time. We used the TMURD, a hospital-based clinical database that provides data for ACEI/ARB monthly prescribing reference (MPR), SCr concentrations, and the eGFR in addition to diagnosis codes and medications, to align baseline characteristics. We also conducted in-depth subgroup evaluations. Despite the merits and innovativeness of the present study, certain limitations are worth noting. First, a larger number of subjects is needed to provide more precise risk estimates. Second, our nonuser group had a much lower underlying CV risk than SGLT2 inhibitor users did. There were significant differences in some baseline characteristics between SGLT2 inhibitor users and nonusers. We did not match more variables at the beginning of the study because of difficulty in reaching a balanced covariate distribution between two groups in the matching of the propensity score. Therefore, we included the following variables in the multivariate regression rather than in the matching process: age and history of CHF, DM, IHD, COPD, and hypertension. Despite our efforts to provide results in a stratified population, we cannot rule out the possibility of unmeasured confounders. Third, the TMURD does not include lifestyle and personal habit information. Finally, compared to that of a randomized controlled trial (RCT), the observational nature of this study focusing on drug epidemiology might have introduced allocation and prescription biases. Although RCTs are the gold standard for demonstrating pharmaceutical impacts, drug epidemiology remains a prevalent approach in medical research. In numerous circumstances, an RCT might not be feasible or appropriate. Such cases include studying potential adverse effects, evaluating drug interactions, investigating genetic predispositions to diseases, and scrutinizing the outcomes of drug overdoses.

In conclusion, in this study of CKD patients (stages 3~5) receiving ACEIs/ARBs who were enrolled in a pre-ESRD program with multidisciplinary teams, SGLT2 inhibitor use was associated with a notably greater incidence of GUTIs, regardless of GUTI history. Moreover, SGLT2 inhibitor users had a significant reduction in the risk of ESRD/dialysis, and SGLT2 inhibitor use was not associated with episodic risks of AKI or subsequent AKD. SGLT2 inhibitor users without a history of CVD had lower CHF, arrhythmia, and AMI rates, although these differences were not statistically significant. A personalized medicine approach, informed by the latest evidence and shared decision-making, will ensure a balance between GUTI episodes and cardiorenal outcomes in patients receiving SGLT2 inhibitors.

## Supplementary Material

Supplementary table.

## Figures and Tables

**Table 1:** Baseline characteristics of SGLT2 inhibitor users and nonusers in patients with chronic kidney disease stages 3-5 who were receiving ACEIs/ARBs

Characteristics	Nonusers, n (%)(N = 820)	SGLT2 inhibitor users, n (%)(N = 205)	p value
Males	508 (62)	127 (62)	1.000
Stage (pre-ESRD)			1.000
3	612 (74.6)	153 (74.6)	
4	164 (20)	41 (20)	
5	44 (5.4)	11 (5.4)	
Age (years) [mean, SD]	71.88 (13.06)	67.55 (11.91)	< 0.001
20-44	25 (3)	11 (5.4)	0.001
45-64	198 (24.1)	52 (25.4)	
65-74	227 (27.7)	88 (42.9)	
75-90	370 (45.1)	54 (26.3)	
Baseline creatinine, mg/dL [mean, SD]	2.45 (1.96)	2.01 (1.05)	0.418
Baseline eGFR, mL/min/1.73 m^2^ [mean, SD]	35.58 (13.18)	40.45 (13.1)	0.016
ACEIs/ARBs, MPR [mean, SD]	0.39 (0.38)	0.57 (0.41)	< 0.0001
≥ 80%	202 (24.6)	88 (42.9)	
40%-80%	99 (12.1)	39 (19)	
< 40%	519 (63.3)	78 (38)	
Within 1 year before index date CCI [mean, SD]	3.44 (2.24)	4.09 (2.14)	0.004
CHA_2_DS_2_-VASc [mean, SD]	3.4 (1.52)	3.68 (1.34)	0.006
0-2	241 (29.4)	39 (19)	0.012
3-5	511 (62.3)	146 (71.2)	
≥ 6	68 (8.3)	20 (9.8)	
History of hospitalization			
AMI	11 (1.3)	4 (2)	0.516
Heart failure	52 (6.3)	27 (13.2)	0.001
Stroke	28 (3.4)	7 (3.4)	1.000
Comorbidities			
Diabetes mellitus	431 (52.6)	180 (87.8)	< 0.001
IHD	266 (32.4)	103 (50.2)	< 0.001
Atrial fibrillation	40 (4.9)	14 (6.8)	0.263
Hyperlipidaemia	422 (51.5)	121 (59)	0.052
PVD	19 (2.3)	7 (3.4)	0.371
COPD	87 (10.6)	32 (15.6)	0.046
CLD	36 (4.4)	9 (4.4)	1.000
Dementia	46 (5.6)	6 (2.9)	0.117
Hypertension	669 (81.6)	181 (88.3)	0.022
Medication use			
Clopidogrel	93 (11.3)	34 (16.6)	0.042
Dipyridamole	56 (6.8)	10 (4.9)	0.309
Warfarin	9 (1.1)	4 (2)	0.329
Loop diuretics	167 (20.4)	46 (22.4)	0.513
Beta-2 blockers	242 (29.5)	99 (48.3)	< 0.001
CCBs	244 (29.8)	59 (28.8)	0.784
Antiplatelet drugs	245 (29.9)	83 (40.5)	0.004
Statins	237 (28.9)	101 (49.3)	< 0.001
NSAIDs	32 (3.9)	10 (4.9)	0.529
Metformin	67 (8.2)	54 (26.3)	< 0.001
Thiazolidinedione	12 (1.5)	11 (5.4)	0.001
Sulfonylureas	76 (9.3)	58 (28.3)	< 0.001
AGIs	35 (4.3)	26 (12.7)	< 0.001
DPP4is	174 (21.2)	68 (33.2)	< 0.001
Insulin	106 (12.9)	40 (19.5)	0.016
Follow-up (year) [mean, SD]	1.56 (1.2)	1.61 (1.22)	

Abbreviations: ACEIs: angiotensin-converting enzyme inhibitors; AGIs: alpha-glucosidase inhibitors; AMI: acute myocardial infarction; ARBs: angiotensin receptor blockers; CCBs: calcium channel blockers; CCI: Charlson comorbidity index; CHA2DS2-VASc: congestive heart failure, hypertension, age ≥75 years [doubled]; diabetes mellitus, prior stroke or transient ischaemic attack [doubled]; vascular disease, age 65-74 years, female; CKD: chronic kidney disease; CLD: chronic liver disease; COPD: chronic obstructive pulmonary disease; DPP4is: dipeptidyl peptidase 4 inhibitors; eGFR: estimated glomerular filtration rate; IHD: ischaemic heart disease; MPR: monthly prescribing reference; NSAIDs: nonsteroidal anti-inflammatory drugs; PVD: peripheral vascular disease; SD: standard deviation; SGLT2: sodium‒glucose cotransporter 2; SMD: standardized mean difference.

**Table 2:** The incidence (per 100 PY) and risk of subsequent events of SGLT2 inhibitors in patients with chronic kidney disease stages 3-5 who received ACEIs/ARBs and participated in a pre-ESRD program

Group	No. of events	PY	Incidence (95% CI)	Crude HR (95% CI)	p value	Adjusted HR^*^ (95% CI)	p value
Congestive heart failure					0.782		0.345
Nonusers	75	1188	6.31 (4.96-7.91)	1.00 (Ref.)		1.00 (Ref.)	
SGLT2 inhibitor users	18	308	5.84 (3.46-9.23)	0.93 (0.56-1.56)		0.77 (0.44-1.33)	
APE					0.052		0.154
Nonusers	13	1255	1.04 (0.55-1.77)	1.00 (Ref.)		1.00 (Ref.)	
SGLT2 inhibitor users	8	319	2.51 (1.08-4.95)	2.40 (0.99-5.78)		2.02 (0.77-5.29)	
Arrhythmia					0.921		0.397
Nonusers	32	1239	2.58 (1.77-3.64)	1.00 (Ref.)		1.00 (Ref.)	
SGLT2 inhibitor users	8	319	2.51 (1.08-4.94)	0.96 (0.44-2.09)		1.46 (0.61-3.53)	
Acute myocardial infarction					0.458		0.198
Nonusers	26	1240	2.10 (1.37-3.07)	1.00 (Ref.)		1.00 (Ref.)	
SGLT2 inhibitor users	9	319	2.82 (1.29-5.35)	1.33 (0.62-2.84)		1.78 (0.74-4.30)	
Sepsis					0.697		0.506
Nonusers	53	1233	4.30 (3.22-5.62)	1.00 (Ref.)		1.00 (Ref.)	
SGLT2 inhibitor users	15	312	4.80 (2.69-7.92)	1.12 (0.63-1.99)		1.23 (0.67-2.25)	
GUTIs					0.019		0.013
Nonusers	70	1153	6.07 (4.73-7.67)	1.00 (Ref.)		1.00 (Ref.)	
SGLT2 inhibitor users	29	281	10.33 (6.91-14.83)	1.68 (1.09-2.59)		1.80 (1.13-2.86)	
ESRD + dialysis					0.081		0.001
Nonusers	77	1169	6.59 (5.20-8.23)	1.00 (Ref.)		1.00 (Ref.)	
SGLT2 inhibitor users	12	318	3.78 (1.95-6.60)	0.58 (0.32-1.07)		0.35 (0.19-0.66)	
AKI					0.962		0.456
Nonusers	19	1251	1.52 (0.91-2.37)	1.00 (Ref.)		1.00 (Ref.)	
SGLT2 inhibitor users	5	323	1.55 (0.50-3.62)	1.02 (0.38-2.74)		0.67 (0.24-1.91)	
AKD					0.795		0.638
Nonusers	5	1260	0.40 (0.13-0.93)	1.00 (Ref.)		1.00 (Ref.)	
SGLT2 inhibitor users	1	324	0.31 (0.01-1.72)	0.75 (0.09-6.44)		0.59 (0.06-5.41)	

Abbreviations: ACEIs: angiotensin-converting enzyme inhibitors; AKD: acute kidney damage; AKI: acute kidney injury; APE: acute pulmonary embolism; ARBs: angiotensin receptor blockers; CI: confidence interval; ESRD: end-stage renal disease; HR: hazard ratio; PY: person-years; SGLT2: sodium‒glucose cotransporter 2; GUTIs: genitourinary tract infections. Notes: ^*^ Adjusted for the following variables: age and history of heart failure, diabetes mellitus, ischaemic heart disease, chronic obstructive pulmonary disease, and hypertension).

## Abbreviations

ACEIs: angiotensin-converting enzyme inhibitors
ARBs: angiotensin receptor blockers
AMI: acute myocardial infarction
AKI: acute kidney injury
AKD: acute kidney disease
APEs: acute pulmonary embolisms
AGIs: alpha-glucosidase inhibitors
ATC: Anatomical Therapeutic Chemical Classification System
CKD: chronic kidney disease
CCI: Charlson comorbidity index
CVD: cardiovascular disease
CHF: congestive heart failure
CLD: chronic liver disease
CCBs: calcium channel blockers
CV: cardiovascular disease
COPD: chronic obstructive pulmonary disease
CI: confidence interval
DPP4is: dipeptidyl peptidase-4 inhibitors
eGFR: estimated glomerular filtration rate
FDA: Food and Drug Administration
IHD: ischaemic heart disease
NIH: National Institutes of Health
NSAIDs: nonsteroidal anti-inflammatory drugs
PVD: peripheral vascular disease
P4P: Pay for performance
SCr: serum creatinine
SGLT2: sodium‒glucose cotransporter-2
SD: standard deviation
TMURD: Taipei Medical University Research Database
GUTI: genitourinary tract infections
