# Supplementary material for: Investigation of cardiorenal outcomes and incidence of genitourinary tract infection after combined SGLT2 inhibitor and ACEI/ARB use in patients with chronic kidney disease stages 3-5: A real-world retrospective cohort study in Taiwan
Source: Int J Med Sci. 2024 Aug 12;21(11):2109–18. doi: 10.7150/ijms.96969 (PMC11373557; doi:10.7150/ijms.96969)
Supplement: Supplementary file 1 — Supplementary table. [file ijmsv21p2109s1.pdf]

Supplementary material

Supplementary Table 1. Disease diagnosis codes According to the ICD-9-CM, ICD-10-CM, and ATC classifications of medications and reimbursement codes for procedures.

**Table S1** Disease diagnosis codes According to the ICD-9-CM, ICD-10-CM, and ATC classifications of medications and reimbursement codes for procedures		
**Clinical outcomes and comorbidities**	**Definition**	
Acute kidney injury (AKI) stage	AKI is defined as an abrupt decrease in kidney function occurring within 7 days or less after the index date, divided into AKI stages 0, 1, 2, and 3 multiplied by serum creatinine (SCr) levels. AKI stage 0 (SCr < 1.5 mg/dL), AKI stage 1 (SCr 1.5~1.9 mg/dL), AKI stage 2 (SCr 2.0~2.9 mg/dL) , and AKI stage 3 (SCr ≥3.0 mg/dL).	
Acute kidney damage (AKD) stage	AKD is divided into AKD stages 0, 1, 2, and 3 multiplied by the SCr level, describing acute or subacute damage and loss of kidney function for a duration of between 7 and 90 days after the index date. AKD stage 0 (SCr <1.5 mg/dL), AKD stage 1 (SCr 1.5~1.9 mg/dL), AKD stage 2 (SCr 2.0~2.9 mg/dL), and AKD stage 3 (SCr ≥3.0 mg/dL).	
Chronic kidney disease (CKD)	CKD is defined as kidney disease lasting more than 90 days (increased SCr levels, >1.5 mg/dL) after the index date by comparing to baseline SCr levels.	
	**ICD-9-CM**	**ICD-10-CM**
Congestive heart failure	428, 402.11, 402.91, 404.11, 404.13, 404.91, 404.93	I110, I130, I132, I501, I5020, I5021, I5022, I5023, I5030, I5031, I5032, I5033, I5040, I5041, I5042, I5043, I509
Acute pulmonary embolism	518.4	J81.0
Arrhythmia	427	I46, I47, I49

Acute myocardial infarction	410	I21, I22
Urinary tract infection	995, 038	A40, A41
Sepsis	590, 599	N10, N11, N15
End-stage renal disease	585.6	N18.6
	**NHI procedure / device code**	
Dialysis	58001C, 58019C, 58020C, 58021C, 58022C, 58023C, 58024C, 58025C, 58029C, 58002C, 58009A, 58009B, 58010A, 58010B, 58011A, 58011AB, 58011B, 58011C, 58012A, 58012B, 58017B, 58017C, 58026C, 58028C, 58018C, 58027C, 58030B	
Diabetes mellitus	250~250.3, 250.7, 250.4~250.6	E0800, E0801, E08311, E08319, E08321, E08329, E08331, E08339, E08341, E08349, E08351, E08359, E0836, E0839, E0840, E0841, E0842, E0843, E0844, E0849, E0851, E0852, E0859, E08641, E0900, E0901, E09311, E09319, E09321, E09329, E09331, E09339, E09341, E09349, E09351, E09359, E0936, E0939, E0940, E0941, E0942, E0943, E0944, E0949, E0951, E0952, E0959, E09641, E1010, E1011, E1021, E1022, E1029, E10311, E10319, E1036, E1039, E1040, E1041, E1044, E1049, E1051, E1052, E1059, E10610, E10641, E1065, E1069, E109, E1100, E1101, E1121, E1122, E1129, E11311, E11319, E11321, E11329, E11331, E11339,

		E11341, E11349, E11351, E11359, E1136, E1139, E1140, E1141, E1142, E1143, E1144, E1149, E1151, E1152, E1159, E11641, E1165, E1169, E119, E1300, E1301, E1311, E1321, E1322, E1329, E13311, E13319, E13321, E13329, E13331, E13339, E13341, E13349, E13351, E13359, E1336, E1339, E1340, E1341, E1342, E1343, E1344, E1349, E1351, E1352, E1359, E13641, E139
Ischemic heart disease (IHD)	410~414	I20, I21, I22, I24, I25
Atrial fibrillation	427.31	I480, I482, I4891
Hyperlipidemia	272	E780, E781, E782, E783, E784, E785
Peripheral vascular disease (PVD)	443.9, 441~441.9, 785.4	I7100, I7101, I7102, I7103, I711, I712, I713, I714, I715, I716, I718, I719, I739, I77819, I790, I96, Z95820, Z95828
Chronic pulmonary disease (COPD)	490~496, 505, 506.4	J40, J410, J411, J418, J42, J430, J431, J432, J438, J439, J440, J441, J449, J470, J471, J479
Chronic liver disease (CLD)	571.2, 571.4, 571.5, 571.6, 456~456.21, 572.2~572.8	K702, K7030, K7031, K7210, K7211, K7290, K7291, K730, K731, K732, K738, K739, K740, K741, K742, K743, K744, K745, K7460, K7469, K754, K766, K767, K7681
Dementia	290	F0150, F0151, F0390, F05
**Medications**	**ATC code**	
Clopidogrel	B01AC04	
Dipyridamole	B01AC07	
Warfarin	B01AA03	

Loop diuretics	C03CA01, C03CA02, C03CA03, C03CA04	
Beta-2 blockers	C07	
CCBs	C08	
Antiplatelet drugs	B01AC	
Statins	C10AA	
NSAIDs	M01AA, M01AB, M01AC, M01AE, M01AG, M01AH, M01AX	
Metformin	A10BA	
Thiazolidinedione	A10BG	
Sulfonylureas	A10BB	
Alpha-glucosidase inhibitor (AGIs)	A10BF	
DPP4is	A10BH, A10BD07, A10BD08, A10BD10, A10BD11	
Insulin	A10A	
ACEIs/ARBs	C09	

ICD-9-CM, International Classification of Diseases, Ninth Revision, Clinical Manifestations; ICD-10-CM, International Classification of Disease, Tenth Revision, Clinical Manifestations; ATC, Anatomical Therapeutic Chemical Classification System; NHI, National Health Institutes; CCBs, calcium channel blockers; NSAIDs, non-steroidal anti-inflammatory drugs; DPP4is, dipeptidyl peptidase 4 inhibitors.
